# Asthma medication adherence and exacerbations and lung function in children managed in Leicester primary care

**DOI:** 10.1038/s41533-022-00323-6

**Published:** 2023-03-25

**Authors:** Razi Paracha, David K. H. Lo, Ursula Montgomery, Louise Ryan, Vivek Varakantam, Erol A. Gaillard

**Affiliations:** 1 University Hospitals of Leicester NHS Trust, Leicester, UK; 2 Department of Paediatric Respiratory Medicine, University Hospitals of Leicester NHS Trust., Leicester, UK; 3 Department of Respiratory Sciences, College of Life Sciences, NIHR Biomedical Research Centre (Respiratory theme), University of Leicester, Leicester, UK; 4 The Central Surgery, Leicester, UK; 5 The Croft Medical Centre, Leicester, UK

**Keywords:** Paediatric research, Outcomes research

## Abstract

Poor adherence to asthma preventer medication is associated with life-threatening asthma attacks. The quality and outcomes framework mandated primary care annual asthma review does not include adherence monitoring and the effect of poor adherence on lung function in paediatric primary care patients is unknown. The aim was to investigate the link between inhaled corticosteroid (ICS) adherence and spirometry, fraction of exhaled nitric oxide (FeNO) and asthma control in asthmatic school-age children in this cross-sectional observational study involving three Leicestershire general practices. Children 5–16 years on the practice’s asthma registers, were invited for a routine annual asthma review between August 2018 and August 2019. Prescription and clinical data were extracted from practice databases. Spirometry, bronchodilator reversibility (BDR) and FeNO testing were performed as part of the review. 130 of 205 eligible children (63.4%) attended their review. Mean adherence to ICS was 36.2% (SEM 2.1%) and only 14.6% of children had good adherence (≥75% prescriptions issued). We found no differences in asthma exacerbations in the preceding 12 months between the adherence quartiles. 28.6% of children in the lowest and 5.6% in the highest adherence quartile had BDR ≥ 12% but this was not statistically significant (*p* = 0.55). A single high FeNO value did not predict adherence to ICS. Adherence to ICS in children with asthma in primary care is poor. The link between adherence to ICS and asthma exacerbations, spirometry and FeNO is complex but knowledge of adherence to ICS is critical in the management of children with asthma.

## Introduction

UK children with asthma have the highest rate of severe asthma attacks of any high-income country in Europe^1^. Over 150,000 children have severe asthma attacks each year^2^, and 26,000 require hospital admission^3^, the equivalent of one child being admitted to a UK hospital every 20 min. These figures have changed little over the last two decades and this is identified as a health priority in the 2019 NHS long-term plan^4^.

Most UK children with asthma are managed in primary care. Asthma management in primary care is largely symptoms-based^5^ and relies on the Royal College of Physician three questions (RCP3Q) symptom score, which is less useful in children^6^. Importantly, only a third of patients receive an adequate asthma review in primary care^7^ and reviews frequently do not involve formal adherence monitoring.

Adherence to preventer medication and inhaler technique is often poor^8–10^. A recent systematic review, which included mostly US studies, highlighted the association between poor adherence and a higher risk of severe asthma attacks^11^. In the UK, poor adherence to asthma medications has also been associated with an increased risk of life-threatening asthma attacks as demonstrated in the 2014 National Review of Asthma Deaths (NRAD), where over one-third of patients who died were prescribed less than 25% of their required ICS inhalers^12^.

Both, poor lung function and elevated FeNO are associated with an increased risk of asthma attacks^13,14^. There is no data on the link between adherence to preventer medication and objective measures of lung function and airway inflammation in children managed in UK primary care.

The aim of this study was to investigate the link between adherence to preventer asthma inhaler medication and spirometry, bronchodilator reversibility (BDR) and FeNO results in children aged 5–16 years managed in UK primary care.

## Methods

### Design and setting

This observational cross-sectional study took place across three primary care practices in Leicestershire, UK; between August 2018 and August 2019. These three practices were chosen as they were already part of a local innovative quality improvement Fellowship Programme. Ethical approval was not required.

### Participants

Children aged 5–16 years with a recorded diagnosis of asthma on the practice register and prescribed regular inhaled corticosteroids were eligible for inclusion in the study.

Eligible patients were invited for their routine annual asthma review by telephone call or letter. The project was conducted as a quality improvement project in primary care. Written informed consent was obtained. Each clinical review was conducted in line with established UK asthma guidelines. There was no randomisation and no identifiable data collection and therefore, no Research Ethics Committee approval was required.

### Practice database searches

Searches to identify patients on the asthma register were performed across all three practices in Leicestershire using the SystmOne computer system by the practices themselves. Patient electronic records were interrogated to obtain details of asthma attacks in the previous 12 months and to collect adherence data based on the number of asthma medication prescriptions issued over the previous 12 months.

Asthma attacks were defined as an unscheduled healthcare consultation or any hospital attendance with acute wheezing with or without a prescription of systemic corticosteroids.

### Adherence data

The adherence rate was calculated as the ratio of doses of medication issued and a total number of doses in the intended treatment regimen, expressed as a percentage. This method of adherence measurement has been described in previous studies as a “medication possession ratio”^11^. An adherence ratio of ≥75% was considered to be good adherence^15^. The maximum adherence ratio was capped at 100%.

### Asthma reviews

All asthma reviews included an assessment of asthma symptom control, checking of inhaler technique, an update of the personalised written asthma action plan, and lung function testing.

Regular asthma medications were not withheld on the day of review.

### Asthma control

During the face-to-face consultation, parents and the child were asked to complete an asthma control test (ACT)^16^ if ≥12 years old and the Children’s cACT^17^ if <12 years. A score of <20 for either test was considered to indicate poor control.

### Fractional exhaled nitric oxide

The fraction of exhaled nitric oxide (FeNO*)* was measured using a near-patient electrochemical analyser (NIOX VERO®, Circassia Group, Oxford, UK). FeNO was always tested prior to spirometry. The value taken was from the first successful attempt that achieved the sustained flow rate required^18^. A FeNO value ≥35 ppm was taken as the cut-off value for abnormally raised FeNO^19^.

### Spirometry

Spirometry was performed by the quality improvement project fellow using a MicroLab Mk8 flow turbine spirometer (CareFusion, San Diego, CA, USA). Forced expiratory manoeuvres were performed according to American Thoracic Society and European Respiratory Society (ATS/ERS) standards^20^. BDR testing was performed after administering 400 micrograms of salbutamol inhaler via a spacer device. BDR was tested in all patients performing spirometry. Lung function parameters were expressed as percentage predicted for FEV_1_ and FVC, and as the absolute percentage for FEV_1_/FVC. The Global Lung Initiative (GLI) 2012 reference equations were used^21^.

### Data analysis

Statistical analyses were performed using IBM SPSS Statistics for Windows (Version 24.0. Armonk, NY: IBM Corp) and GraphPad Prism version 7.00 for Windows (GraphPad Software, USA, www.graphpad.com).

Continuous variables were compared using unpaired *t*-tests for parametric data, and Kruskal–Wallis and Wilcoxon rank-sum tests for non-parametric data. Chi-squared tests were used for count data.

All statistical tests were performed at the alpha = 5% level.

### Reporting summary

Further information on research design is available in the Nature Research Reporting Summary linked to this article.

## Results

The 3 primary care practices were of similar size (registered patients ranging from 8200 to 9200), and in the 6th, 9th and 10th deciles of deprivation (Table 1). The first decile represents the most deprived areas, and the tenth is the least deprived areas^22^.

**Table 1:** Practice demographics.

	Registered patients	Decile of deprivation index	White %	Asian %	Black %	Other %	Mixed %
Practice 1	8877	9th	62.1	31.7	1.5	2.2	2.5
Practice 2	9254	10th	56.2	37.3	1.5	2.6	2.4
Practice 3	8276	6th	91.4	5.1	0	1.3	2.2

**Fig. 1:**
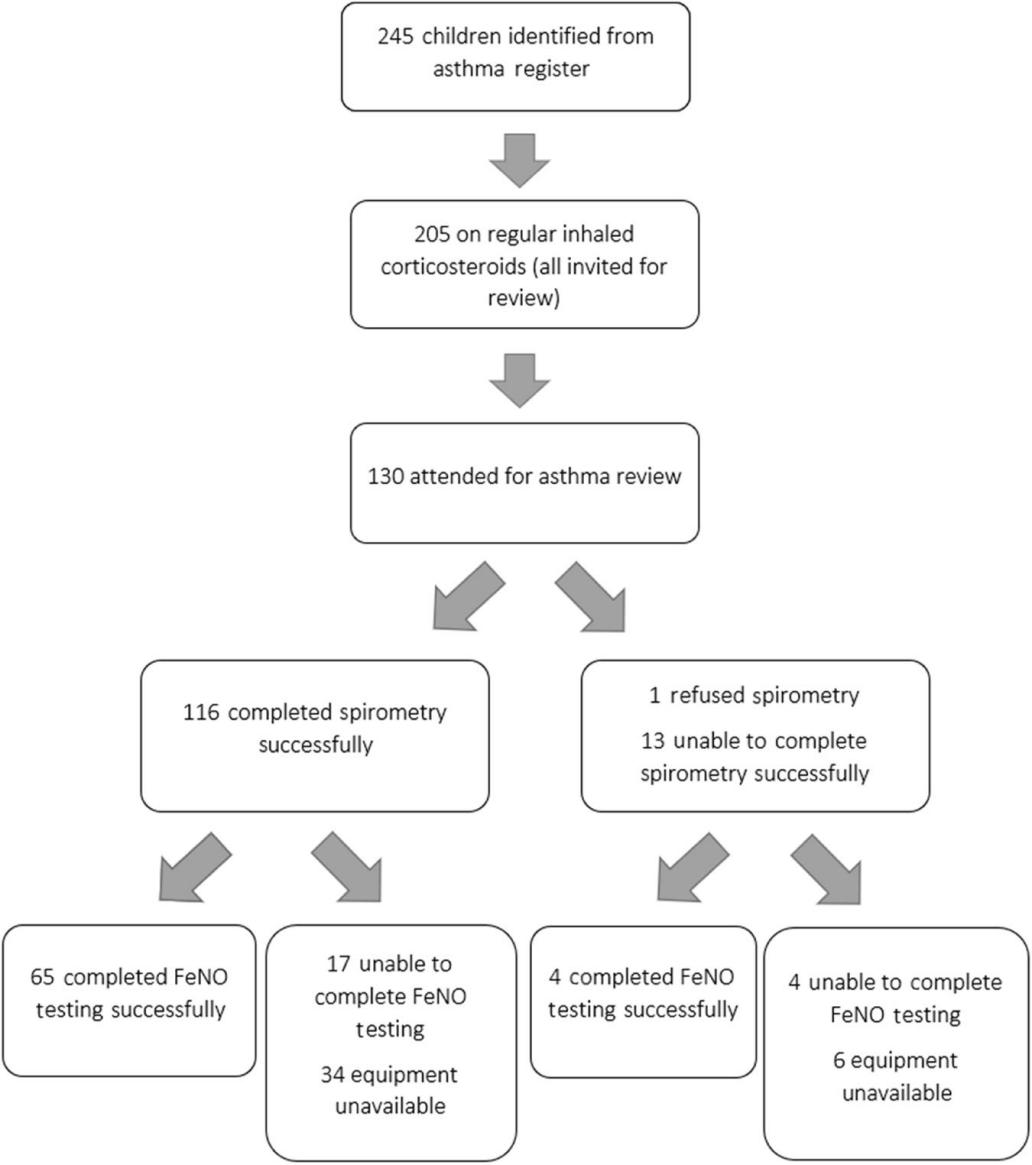
Eligibility and follow-up of children with asthma. Children were invited to attend for an asthma review at their general practice that included spirometry, bronchodilator reversibility and fraction of exhaled nitric oxide (FeNO) testing.

We identified 245 children with an asthma diagnosis code. Of these, 205 were on regular inhaled corticosteroids as part of their asthma treatment and all were invited for a review. 130 out of 205 (63%) attended. Spirometry was attempted in all children, and useable data were obtained from 116 (89%). FeNO equipment was not available at the start of our study but was available for 96 out of 130 children. FeNO was successful in 65 (68%) children (Fig. 1).

Mean (SEM) adherence in the 205 children eligible for participation was found to be 36.2% (2.1%). Only 14.6% of patients had a target adherence rate of ≥75% (quartile 4) (Table 2).

**Table 2:** Patient characteristics and clinical information.

All patients invited for review (*n* = 205)	
Number of males (%)	116 (56.6%)
Median age (IQR)	10 (8–13)
*Ethnicity (%)*	
White	92 (44.9)
Black	9 (4.4)
Asian	72 (35.1)
Other/mixed	32 (15.6)
*Adherence*	
Quartile 1–0 to 24%	74 (36.1)
Quartile 2–25 to 49%	72 (35.1)
Quartile 3–50 to 74%	29 (14.1)
Quartile 4–75 to 100%	30 (14.6)
*All patients attending (n* *=* *130)*	
Males (%)	79 (61)
Median age (IQR)	9 (8 to 12)
*Adherence*	
Quartile 1–0 to 24%	38 (29.2)
Quartile 2–25 to 49%	49 (37.7)
Quartile 3–50 to 74%	22 (16.9)
Quartile 4–75 to 100%	21 (16.2)
*Median asthma control score (IQR)*	
CACT	21.5 (19–24)
ACT	20.0 (16.75–23)
Number of patients with ACT/cACT <20 (%)	49 (37.7%)
Mean FEV_1_ % Predicted (SEM)*	93.3 (1.17)
Mean FEV_1_ *z*-score (SEM)*	−0.56 (0.10)
Mean FEV_1_/FVC (SEM)*	0.90 (0.01)
Mean FEV_1_/FVC *z*-score (SEM)*	0.45 (0.13)
Number of children with BDR ≥ 12% (%)*	21 (18.1%)
Median FeNO (IQR)**	38 (13–56)
Number of children with FeNO ≥ 35 ppb (%)	36 out of 65 (55.1%)

	Adherence quartile				
	Quartile 1 (0–24%) *N* = 38	Quartile 2 (25–49%) *N* = 49	Quartile 3 (50–74%) *N* = 22	Quartile 4 (75–100%) *N* = 21	*P* value
Median ACT (IQR)	21 (18.5–23.5)	20 (15.5–22.5)	19(17–24)	17 (10.5–21)	0.381
Median CACT (IQR)	21 (19–24)	21 (17–24)	21 (19–23)	23 (16–24)	0.972
Mean FEV_1_ % Predicted (SEM)^a^	93.9 (2.57)	91.3 (1.85)	95.1 (2.49)	95.1.8 (2.70)	0.560
Mean FEV_1_ *z*-score (SEM)	−0.52 (0.21)	−0.72 (0.15)	−0.40 (0.20)	−0.41 (0.22)	0.544
Mean FEV_1_/FVC (SEM)^a^	0.90 (0.01))	0.90 (0.01)	0.88 (0.02)	0.93 (0.01)	0.306
Mean FEV_1_/FVC *z*-score (SEM)^a^	0.39 (0.29)	0.57 (0.22)	−0.00 (0.29)	0.80 (0.24)	0.301
Number of children with BDR ≥ 12% (%)^a^	8/28 (28.6%)	10/48 (20.8%)	2/22 (9.1%)	1/18 (5.6%)	0.143
Median FeNO (IQR)^b^	46.5 (17–56)	46 (10–75.75)	18 (12.25–39.5)	36 (14–48)	0.352
Number of children with FeNO ≥ 35 ppb (%)^b^	12/18 (66.7%)	15/28 (53.6%)	4/10 (40%)	5/9 (55.6%)	0.589

^a^Spirometry and BDR data available from 116 children.

^b^FeNO from 65 children.

Forty-nine patients out of 130 (37.7%) had poor control according to their ACT/cACT score. 18% had significant bronchodilator reversibility on spirometry and 55% of the total patients had raised FeNO > 35 ppb.

### Relationship between adherence, spirometry, FeNO and asthma control

Lung function, FeNO and asthma control data for the 130 children attending for review are shown in Table 2.

**Fig. 2:**
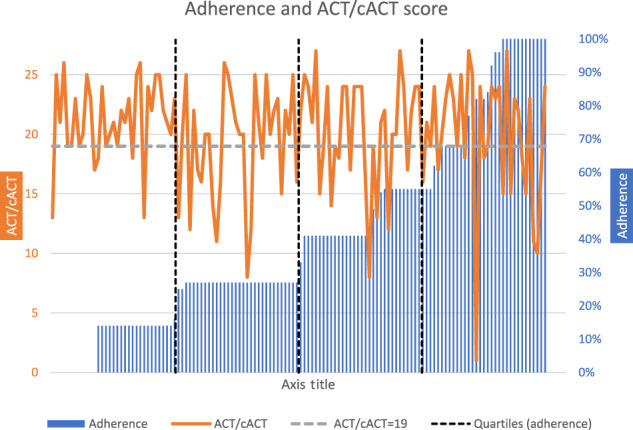
Relationship between adherence and ACT/cACT score plotted for all 130 patients each blue bar represents the adherence of one patient. The grey dotted horizontal line represents an ACT/cACT score of 19. A score <20 represents poor symptom control. The black dotted vertical lines represent quartiles of adherence.

The relationship between adherence and asthma symptom scores is plotted for each patient in Fig. 2.

We found no significant differences in symptom scores, FEV_1_, FEV_1_/FVC, or FeNO between the different adherence categories (Table 2).

There was a trend towards fewer children with a positive BDR ≥ 12% with increasing adherence (*p* = 0.143). In total, 28.6% of children with the poorest adherence (Q1) had BDR > 12% compared to only 5.6% of children with the best adherence (Q4), however, this did not reach statistical significance (*p* = 0.055).

### Relationship between ICS adherence, ethnicity, SABA usage and asthma exacerbations

Seventeen children (8%) had had at least one asthma exacerbation in the previous year. We found no difference in the mean number of attacks between children in different adherence quartiles. We found a significant difference in the number of SABA prescriptions between ICS adherence quartiles, with the fewest number of SABAs prescribed to children with the lowest ICS adherence (Table 3).

**Table 3:** Relationship between ICS adherence with SABA usage and attacks over the 12-month observation period in all children invited for a review (*n* = 205).

	Preventer adherence quartiles				*P* value
	Quartile 1 (0–24%)	Quartile 2 (25–49%)	Quartile 3 (50–74%)	Quartile 4 (75–100%)	
Mean number of attacks (SEM)	0.08 (0.03)	0.06 (0.03)	0.17 (0.07)	0.10 (0.07)	0.362
Mean number of SABA inhalers/year (SEM)	1.55 (0.25)	4.15 (0.37)	4.66 (0.77)	4.20 (0.53)	<0.001

We found no significant differences in adherence rates between children from different ethnic backgrounds (Table 4).

**Table 4:** Relationship between ICS adherence and ethnicity in all children invited for a review (*n* = 205).

	Ethnicity				*P* value
	White	Black	Asian	Other/mixed	
Mean adherence (SEM)	0.36 (0.03)	0.36 (0.10)	0.39 (0.04)	0.30 (0.05)	0.615

## Discussion

Several previous studies and a systematic review reported low adherence to asthma-preventer inhaler medication in children followed up in primary care^8–11,23^. The mean adherence of 36.4% in our cohort of children was also low, and >75% adherence in only 14.6% of patients. A target adherence rate of ≥75% was used as this has been shown to significantly improve asthma control^15,24,25^. The aims of asthma management are to achieve good symptom control and minimise the risk of future asthma attacks and identify the lowest dose of inhaled corticosteroids needed to achieve these goals^5,26^. Although many patients report “good symptom control”, there is discordance between the symptoms reported and objective data^11,27^. Previous studies have reported the connection between low lung function and higher morbidity^13,28,29^.

Our study offers novel insights into the relationship between preventer medication adherence, asthma control and objective measures of lung function and airway inflammation. The complex link between ICS adherence and asthma exacerbations, spirometry, BDR and FeNO is highlighted in this study. Overall, we found no significant differences in lung function, FeNO and ACT scores between the adherence quartiles. This finding highlights that children with asthma are a heterogeneous group. Objective measures of lung function and airway inflammation; combined with the knowledge of asthma symptom control and adherence, provide a much more granular picture to allow the formulation of an effective management strategy. This strategy can then be tailored to the needs of the individual child, providing truly personalised medicine. This is not possible when relying on asthma symptom control alone.

BDR, for example, is a marker of airway lability and is associated with poor asthma control^30^. Nearly 30% of children in our lowest adherence quartile showed BDR > 12%, more than in the highest adherence quartile (*p* = 0.055). In fact, 20 of 82 children (24.4%) with <50% adherence demonstrated bronchial lability. These children have active asthma and are likely undertreated, and these patients require intervention towards better adherence. This is also reflected in the finding of the highest median FeNO values in the lowest two quartiles.

In contrast, most children in the lower two adherence quartiles had good symptom control, no significant BDR, and minimal or no use of preventer medication and these children either do not have asthma or do not have active asthma. With evidence of good lung function, these children would merit a trial of formally stopping ICS treatment (step-down treatment).

Only three of 41 children with adherence ≥50% showed BDR ≥ 12%, showing that airway lability is reduced with regular ICS treatment. Children with significant BDR despite regular ICS should be considered for a trial of long-acting beta-agonist treatment (step-up treatment).

A number of children in the highest adherence quartile, indeed a number of these picking up 100% of their prescriptions, remained uncontrolled. We do not know whether these children actually took their inhalers as directed, but once treatment has been escalated to moderate dose ICS plus LABA and control remains poor, these children warrant referral to a specialist paediatric asthma service.

NICE recommends the use of spirometry to support asthma monitoring and management^19^. Our study shows that using spirometry and BDR in cases where baseline spirometry is abnormal i.e., where FEV_1_ and/or FEV_1_/FVC is/are below the lower limit of normal (GLI reference) identified the 18.6% of children with significant BDR, most of whom were in the lower two adherence quartiles. These children have reactive airway disease and are at risk of an asthma attack. This would also strengthen the education of families and children with asthma by showing objective evidence of poor control in the form of lung function tracings.

During the asthma reviews, the adherence rate, asthma control score and objective testing results were all discussed with the patient and parent. A targeted management plan was then formulated. If poor disease control was associated with poor adherence, then treatment was not stepped up and adherence counselling was carried out instead (with follow-up). Similarly, if disease control was good despite poor adherence, treatment could be stepped down. An alternative diagnosis was considered for those with normal objective testing and ongoing symptoms. There is also an opportunity to identify patients with high BDR or FeNO with good subjective symptom control, as these patients may be at risk of severe exacerbations with uncontrolled active disease.

The areas included in the study have a significant minority ethnic population (especially South Asian), and comparing adherence between ethnic groups found no significant difference. Ethnic disparities in the use of asthma controller medication have been reported^31,32^. We found no differences in the adherence to ICS between Black, Asian and White children in our study, suggesting that the reasons for non-adherence are independent of ethnicity.

Accurate prescription data was collected for the 12 months immediately prior to the consultation and respiratory testing. Prescription data gives a much more objective measure of adherence than subjective patient/parental reporting (precluding recall bias)^33^. Outcome measures were also carried out with the validated ACT/cACT questionnaires and objective testing with spirometry and FeNO. This gives us better data than other database studies in which there is little data about asthma severity. In our searches, no similar studies were found in primary care relating adherence to objective testing.

As asthma is a difficult diagnosis, especially in children, we attempted to include patients who may not have been coded correctly on the primary care practice database by including children who had been prescribed 2 or more SABA or preventer inhalers over the past year due to wheeze (and no other established respiratory diagnosis).

Attenders may be a self-selected population and may be more proactive with their disease management. The cohort attending for review had higher adherence compared to those who did not (Table 2). Additionally, the patient population may be a very heterogenous group e.g., quartile 1 might include dormant asthma, those misdiagnosed, as well as patients who are extremely non-adherent. This may contribute to the complex relationships in the data above.

The medication possession ratio is an indirect measure of adherence. This does not tell us if the medication was actually used or properly administrated. Although, this is a pragmatic approach that can be used in current clinical practice without investment into additional measures such as “smart” inhalers. There is evidence that healthcare database information can provide high concordance with other accurate and objective methods such as weighing inhalers or electronic monitoring^34,35^.

Significant challenges exist in adopting lung function testing in primary care.

FeNO equipment was not available during the first part of the study and the test was therefore only available for 69% (90 out of 130) of the study population. There are no previous data that allowed us to perform a meaningful power calculation, and the power of this study is limited by the number of patients, especially with the small number of patients with successful FeNO testing. Raised FeNO is associated with classical, steroid-responsive, type-2 airway inflammation. Raised FeNO is also present in children with other atopic diseases, such as allergic rhinitis and eczema. Due to the cross-sectional observational nature of this study, we cannot be sure that the raised FeNO observed in some patients are due to uncontrolled airway type-2 inflammation. High FeNO values ≥ 35 ppb were observed in all 4 adherence quartiles.

We found no published evidence showing the relationship between adherence and objective testing in children in primary care.

Low mean adherence in our population was consistent with that reported in other studies^11,36,37^. This continues to be extremely poor and shows significant room for improvement in addressing poor outcomes for asthmatic children in the UK.

SABA prescriptions increased with increasing preventer adherence. This has been demonstrated previously^36^ and suggests a complex relationship. Inhaler use may be self-regulated and those with increasing symptoms may be increasingly adherent to preventers while also needing more SABA, suggesting symptom control is still inadequate.

Previous studies examine the relationship between adherence and asthma exacerbations^36–38^, but none have established the relationship between adherence and objective measures of asthma control. Our data show exacerbations are evenly distributed across the quartiles. Worsening spirometry and FeNO can be better indicators of worsening asthma severity^27,39^. Adherence and objective testing together can provide valuable clinical information, but we need a clearer picture of how adherence is related to disease outcomes.

Effective treatments for asthma are available, yet many children’s asthma still remains uncontrolled^27^. Factors such as trigger identification, comorbidities and asthma phenotype, as well as clinician and sociodemographic factors, play an important role^40^. Poor adherence to ICS treatment is an important contributory factor to poor asthma control that can be fairly easily identified, although changing family and child behaviour can be challenging^41,42^. However, the identification of poor adherence is an important first step.

Although the relationships between adherence and indicators of asthma control may be complex, prescription data can provide useful information during primary care consultations. Clinically a practitioner would be able to use adherence information to better target changes in treatment. A child with suboptimal asthma control would need different interventions depending on whether the medication adherence is adequate or not, as stepping up treatment does not address the infrequent use of the medication. The method we have used to review adherence data is instantly accessible to GPs using the SystmOne computer system (currently widely used in the UK), and can easily be accessed during asthma review consultations within the appointment time.

We know that misdiagnosis of asthma in children is common^43,44^. Large numbers of children are over-diagnosed with asthma and these children would not be expected to respond to asthma-preventer medication; hence the poor adherence. This highlights the need for more objective testing to confirm the diagnosis in children.

Considering that patient-reported adherence and control can be very inaccurate, this also supports the increasing role of routine objective testing in primary care. Patients can perceive that their asthma is well controlled, when in fact, objective testing proves otherwise. NICE asthma guideline recommendation for using objective testing does pose a significant challenge with regard to training and time constraints in primary care. In practice, any patient with poor symptom control or abnormal objective testing should have confirmation of the diagnosis and a structured review and follow-up plan until control is achieved^40^.

In summary, more data is needed to establish the relationship between adherence to asthma medication and asthma control (not just exacerbations). The heterogeneity of the patient populations may pose a challenge as it is difficult to discern those who are taking less medication because their symptoms are adequately controlled, from those who do not adhere to their medication regimen resulting in worsening disease control. The impact of reduced adherence and clinical management will be very different in both these groups.

## Supplementary information

Reporting Summary

## Data Availability

The data that support the findings of this study are openly available in Figshare at 10.6084/m9.figshare.21598836.v1.
